# Colloidal few layered graphene–tannic acid preserves the biocompatibility of periodontal ligament cells

**DOI:** 10.3762/bjnano.16.51

**Published:** 2025-05-20

**Authors:** Teissir Ben Ammar, Naji Kharouf, Dominique Vautier, Housseinou Ba, Nivedita Sudheer, Philippe Lavalle, Vincent Ball

**Affiliations:** 1 INSERM UMR_S 1121, CNRS EMR 7003, Université de Strasbourg, Biomaterials and Bioengineering, Centre de Recherche en Biomédecine de Strasbourg, 1 rue Eugène Boeckel, Strasbourg F-67000, France; 2 Blackleaf, Illkirch-Graffenstaden, 67400, France; 3 Faculté de Chirurgie Dentaire, Université de Strasbourg, 8 rue Sainte Elisabeth, 67000 Strasbourg, France; 4 Institut de physique et chimie des Matériaux de Strasbourg (IPCMS), Centre National de la Recherche Scientifique UMR 7504, 23 rue du Loess, 67034 Strasbourg Cedex 2 BP43, France

**Keywords:** antioxidant properties, biocompatibility, dental applications, few layered graphene–tannic acid biocomposite (FLG–TA), periodontal ligament cells (PDL)

## Abstract

Dental diseases pose a global health concern. In addition to medication and care, the use of biocompatible and even bioactive dental materials can contribute to global oral health. Among such materials, nanomaterials begin to be used. In this context, the incorporation of graphene-based materials into dental biomaterials could offer advantages such as increased mechanical strength. Nevertheless, biocompatibility issues still hinder their adoption. In this study, a biocomposite of few-layered graphene and tannic acid (FLG–TA) was synthesized through a straightforward, bio-based methodology. Physicochemical characterizations elucidated the structural and morphological attributes of the biocomposite. By incorporating antioxidant TA molecules onto the FLG surface, the biocomposite dynamically mitigated reactive oxygen species, demonstrating no cytotoxicity to periodontal ligament cells up to 200 µg·mL^−1^ while promoting cellular adhesion and maintaining chromatin integrity. Overall, because of its favorable biocompatibility FLG–TA holds promise as a novel biomaterial for dental applications.

## Introduction

Dental diseases remain a global health challenge [1]. Dental biomaterials are crucial in both therapeutic and preventive strategies, with nanotechnology emerging as a transformative approach to enhance their efficacy [2]. Actually, incorporating nanomaterials into dental biomaterials has already offered advantages like enhanced tissue regeneration, increased mechanical strength of composites, and improved sealing of filler materials [3]. Graphene-based materials (GBMs) stand out for their potential in dentistry due to their high specific surface area, mechanical strength and adaptability for biological and chemical modifications [4–5]. For instance, a previous study demonstrated that incorporating 2% (w/w) graphene oxide into a resin-modified glass ionomer cement significantly enhanced the flexural strength of the material, thereby improving its overall mechanical properties [6]. Additionally, another study investigated the effects of adding graphene oxide nanoplatelets (GONPs) to Portland cement. It was shown that the addition of 1 wt % GONPs improved surface microhardness without compromising biocompatibility [7]. In another work, the formulation of an injectable calcium phosphate cement–chitosan–graphene oxide (GO) composite was found to be effective. This composite fostered the proliferation of human dental pulp stem cells [8]. Despite these promising findings, the clinical use of GBMs in dental practice remains limited. Concerns about their biocompatibility and potential cytotoxic effects continue to hinder their adoption [3–4]. In numerous studies, GO has frequently been the focus of research concerning its incorporation into dental biomaterials. However, its production typically involves harsh chemical treatments, which can introduce defects and impurities. These alterations may negatively affect its desirable properties and compromise its biocompatibility. Previous assessments have demonstrated that the cytotoxicity of these products is mainly determined by the presence of impurities in the graphene structure [9]. Among other GBMs, few-layer graphene (FLG), synthesized through liquid-phase exfoliation (LPE), has emerged as a particularly advantageous option [10–11]. LPE is highly regarded for its simplicity, scalability, and effectiveness in yielding high-quality graphene [10–11]. The ability to produce large quantities of FLG with minimal structural defects makes this method particularly well-suited for industrial applications, including those in the dental field [4,12]. A key aspect of LPE is the use of surfactants to prevent the reaggregation of exfoliated graphene layers [11]. Various surfactants have been employed. For instance, sodium dodecylbenzene sulfonate and sodium cholate have been reported to be able to produce stable graphene colloidal dispersions [13]. However, these synthetic surfactants often raise concerns about toxicity and environmental impact, limiting their practical use [10–11,14]. This has sparked growing interest in more sustainable and biocompatible alternatives. Tannic acid (TA), a naturally derived polyphenolic compound, has shown promise as a bio-derived surfactant for graphene exfoliation [14–15]. Recent studies point to a potential synergistic effect between TA and GBMs [15–17]. TA not only stabilizes graphene sheets in aqueous solutions, preventing their aggregation by interacting with carbon layers at a molecular level [14], but also could impart additional bioactive properties, particularly antioxidant effects [16–18]. Bioactive antioxidants reduce oxidative stress by inhibiting unstable oxygen radicals [17]. Oxidative stress occurs when reactive oxygen species (ROS) damage cellular components because of an imbalance in the normal redox state of cells [17,19]. ROS, which include superoxide radicals, hydroxyl radicals, and hydrogen peroxide, are natural byproducts of the oxidative metabolism. Mammalian cells maintain balanced ROS levels for homeostasis and cellular proliferation. However, excess ROS creates an electron imbalance that triggers continuous electron transfer reactions, leading to oxidative stress [17].

Studies have shown that TA exhibits significant antioxidant properties by suppressing hydroxyl radical formation and neutralizing both superoxide anion radicals and hydrogen peroxide. However, its antioxidant efficacy is concentration-dependent. While it shows antioxidant properties at lower concentrations, TA can act as a prooxidant at higher concentrations. Under these conditions, it binds to metal ions, potentially increasing oxidation and causing damage to biomolecules, especially DNA [17]. Doping GBMs with bioactive molecules like TA represents a potentially effective strategy for enhancing their intrinsic antioxidant properties. Herein we report the synthesis of a biocompatible FLG–TA colloidal system with dual functionality. First, we examine whether TA enables the formation of a stable FLG–TA colloid; thus, various physicochemical characterizations were employed to determine structure and surface functionalities. Second, by employing TA, we aim to develop graphene layers rich in polyphenols to serve as a bioactive interface for cellular interactions. Given that TA possesses potential antioxidant properties, we assessed the in vitro free radical scavenging capability of the FLG–TA system. Ultimately, we examine if these antioxidant features play a role in preserving the biocompatibility of periodontal ligament cells (PDL). As our ultimate objective is to incorporate the composite into dental cements, various biological assays were conducted to evaluate the biocompatibility with PDL cells. Importantly, our strategy indicates that the modified attributes of FLG–TA enhance its suitability for dental biomaterials, thereby paving the way for safer and more effective dental applications.

## Results and Discussion

### Synthesis of FLG–TA results in a few-layered structure with few defects

**Figure 1:**
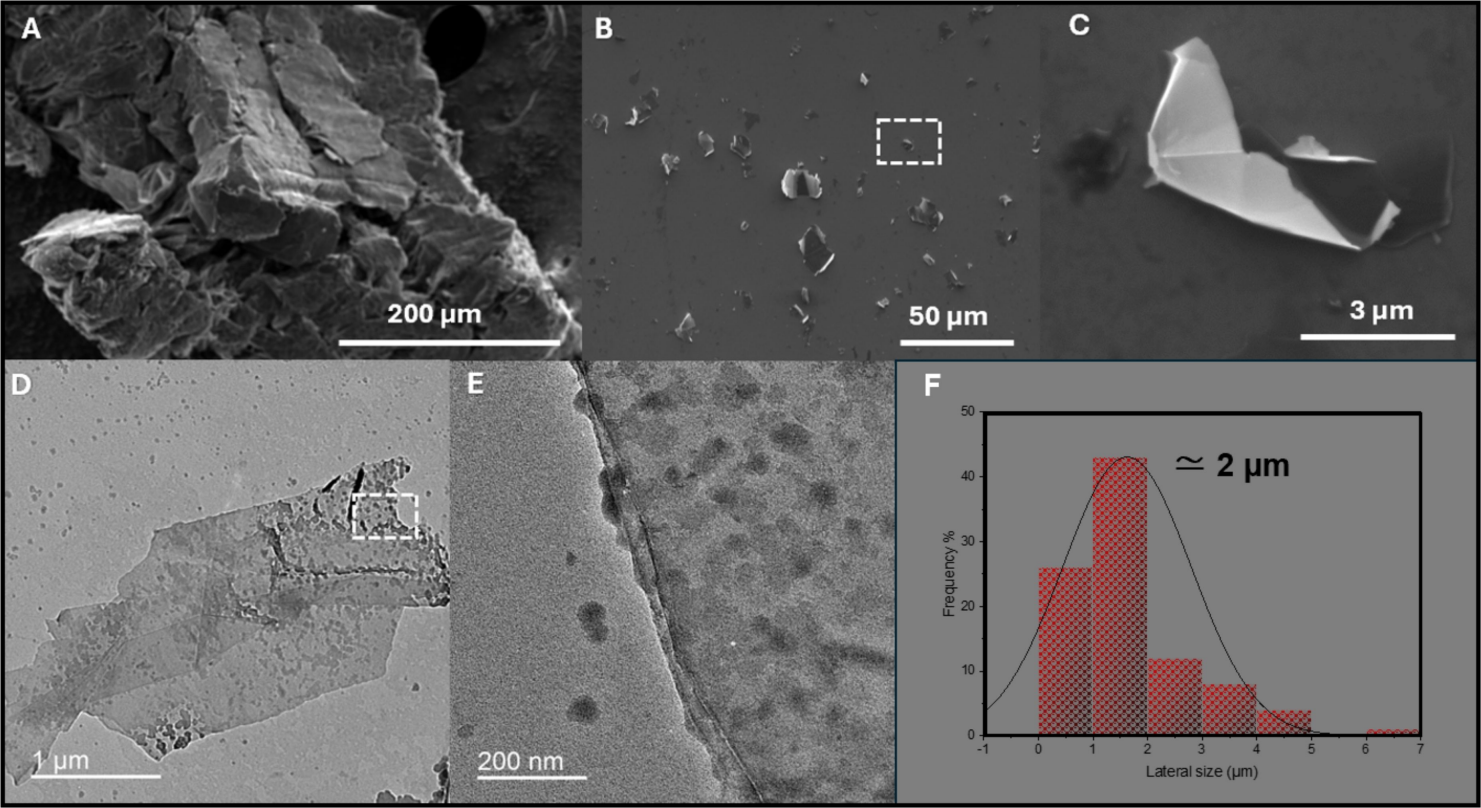
Scanning electron microscope (SEM) micrographs of initial graphite (A) and few-layer graphene (FLG) sheets obtained after the exfoliation of graphite assisted by TA (B,C). Image (C) originates from the marked box in image (B). Transmission electron microscopy (TEM) micrographs of exfoliated FLG–TA (D,E). Size distribution and average lateral size of FLG sheets (F).

The synthesis process combined graphite and TA in a 10:1 mass ratio in an aqueous medium, followed by probe ultrasonication enhanced by magnetic stirring, resulting in a stable colloid as shown in Figure S3, Supporting Information File 1. The polyphenolic structure of tannic acid, characterized by numerous hydroxy groups, is proposed to potentially form non-covalent interactions, such as π–π stacking, with the carbon network and oxygen atoms of graphite [16,20]. This interaction could exfoliate graphite, resulting in a stable colloidal dispersion of functionalized graphene sheets. The conversion was verified, and the properties of the synthesized colloid were characterized using a complementary set of analytical techniques. SEM analysis reveals differences between the initial graphite and the synthesized FLG–TA colloid (Figure 1A–C). The initial graphite appears as large, tightly packed granules, each being hundreds of micrometers in size (Figure 1A). In contrast, the obtained FLG–TA colloid has a layered structure, and sheets seem to have been peeled off from the graphite surface (Figure 1B,C). Transmission electron microscopy (TEM) micrographs of the flakes’ edges (Figure 1D,E) reveal more in-depth its layered structure, consisting of approximately four layers. Quantitative analysis of SEM and TEM images enabled the determination of the FLG–TA sheets’ average lateral size distribution, presented in Figure 1F, indicating an average lateral size of approximately 2 µm. Note that the determination of the size distribution was attempted by means of dynamic light scattering but was unsuccessful owing to the lack of transparency of the suspensions even after strong dilution. The TEM images also reveal visible granules atop the layers (Figure 1D,E). Additional analysis of these granules (Figure S1B, Supporting Information File 1) shows that they are spherical and that they remodel into clusters on the surface.

**Figure 2:**
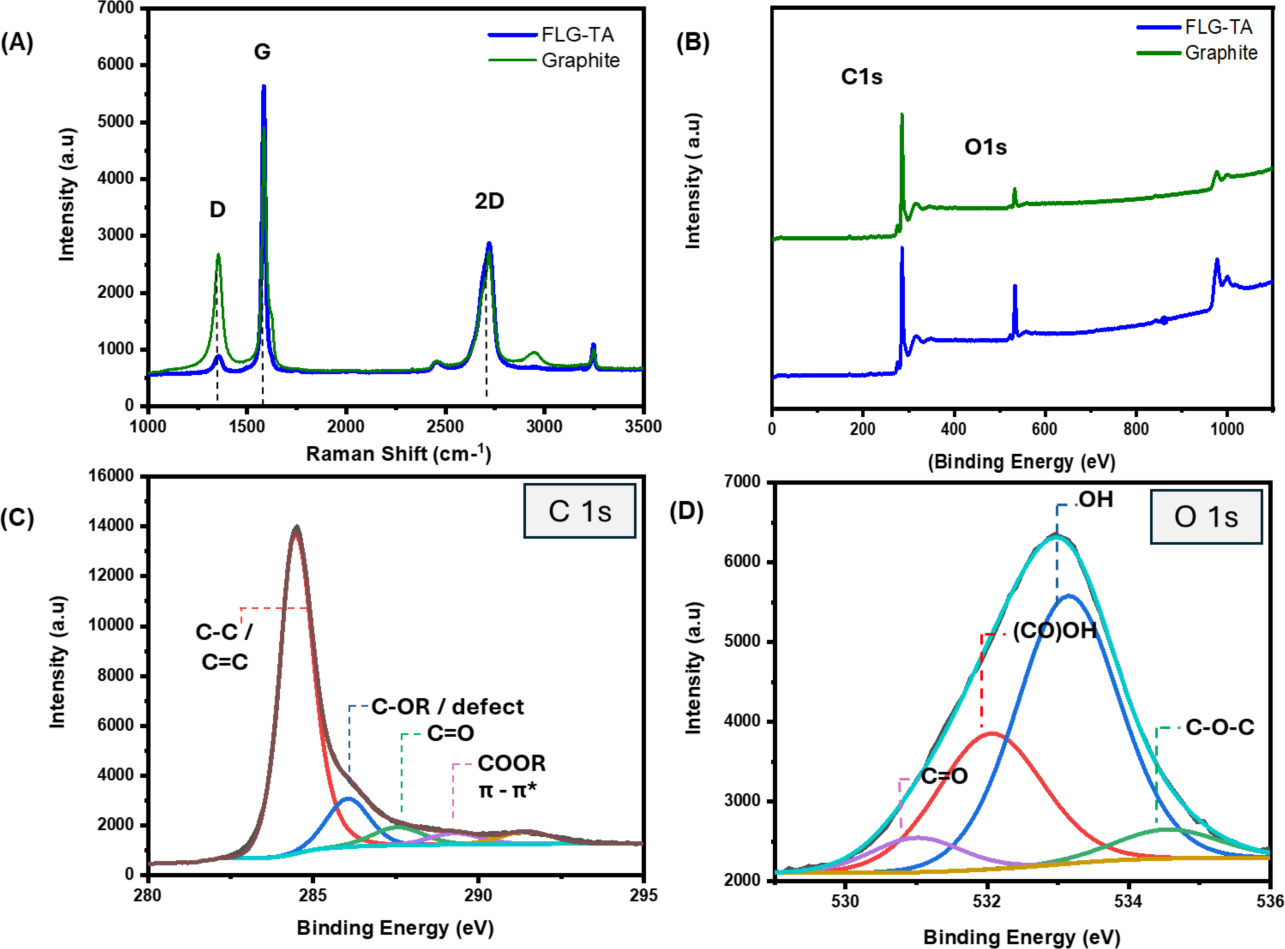
Raman spectra (A) and survey XPS spectra (B) of FLG–TA and initial graphite. Deconvoluted C 1s (C) and O 1s (D) spectra of FLG–TA.

Raman spectra recorded for the initial graphite and the synthesized FLG–TA exhibits typical D, G, and 2D vibration bands centered at 1355, 1583, and 2720 cm^−1^, respectively. It is known that the D band corresponds to structural defects, while the G band relates to the doubly degenerated phonon mode associated with the relative movement of sp^2^-bonded C–C atoms [21]. Figure 2A shows that the *I*_D_/*I*_G_ ratio shifts from 0.54 for initial graphite to 0.16 for FLG–TA. This data indicates FLG–TA has a low defect content, evidenced by its weak D peak. Although the absorption of carbonyl functions in TA molecules might influence the defect ratio for FLG–TA because of the increase in the G peak (Figure S2, Supporting Information File 1, shows the Raman spectrum of TA powder), it remains significantly lower than the ratios reported for alternative synthesis methods in earlier studies [15]. In fact, polyphenolic compounds, such as TA, can act as electron donors [15,22], consequently triggering a change in the G band intensity. This change is likely due to the incorporation of electron donor groups into the graphene structure. In our scenario, the integration of mixing and ultrasonication greatly aided the homogenization process and improved the interactions at the molecular level between the functional groups of tannic acid and the carbon layers of graphene, resulting in defects mainly occurring at the edges rather than within the bulk structure. This low defect ratio, accomplished through the use of TA, offers a straightforward and potentially biocompatible method for producing a high-quality FLG–TA biocomposite suitable for dental applications.

### Arrangement of TA molecules on the surface of graphene confers antioxidant properties

The arrangement of TA molecules on graphene layers and their antioxidant effects were analyzed through three experimental methods. The concentration of TA in the FLG suspension was measured using UV–vis spectroscopy, surface composition changes from graphite to the FLG–TA colloid were analyzed using X-ray photoelectron spectroscopy (XPS), and the antioxidant properties of FLG–TA were evaluated using the DPPH free radical scavenging assay. XPS analysis (Figure 2 and Table S1, Supporting Information File 1) shows a significant increase in the O-to-C ratio, rising from 0.17 in graphite to 0.45 in the FLG–TA material. In addition, the high-resolution C 1s and O 1s spectra of the FLG–TA composite show the presence of carbonyl, ether, and hydroxy groups (Figure 2C,D). The percentage of those moieties in the FLG–TA material increases significantly with respect to unexfoliated graphite (Table S1, Supporting Information File 1), a strong evidence for the incorporation of TA.

After exfoliation, the UV spectra analysis indicated that nearly all of TA used in the preparation adhered to the FLG, with only 15 µg remaining as free TA molecules (from an initial amount of 100 µg) in the filtrate (Figure S3B, Supporting Information File 1). The DPPH scavenging assay reveals differences in TA antioxidant behavior. Free TA molecules in FLG suspension eliminated approximately 90% of free radicals, while FLG–TA films deposited on gold scavenged only 15% of the provided DPPH (Figure S4B0–B2 of Supporting Information File 1). These findings indicate significant oxygen enrichment due to TA adsorption on FLG sheets, supporting earlier Raman spectroscopy results and colloidal stability observations (Figure S3A1, Supporting Information File 1). Both free and adsorbed TA molecules demonstrated antioxidant activity. We assume that the granules observed in TEM micrographs (Figure 1E) are probably TA aggregates that partially retain their antioxidant properties. These findings align with previous studies where TA was reported to form aggregates through intermolecular hydrogen bonds and coat various surfaces [23]. Our results are compatible with a model where adsorbed TA molecules create a partially active antioxidant layer on the FLG surface, maintaining bioactivity while enhancing graphene colloid stability. Unfortunately, the zeta potential of the FLG–TA material in suspension could not be determined owing to uncontrolled heating of the cell during illumination of the black suspension with laser light.

### FLG–TA preserves the metabolic activity of PDL cells

**Figure 3:**
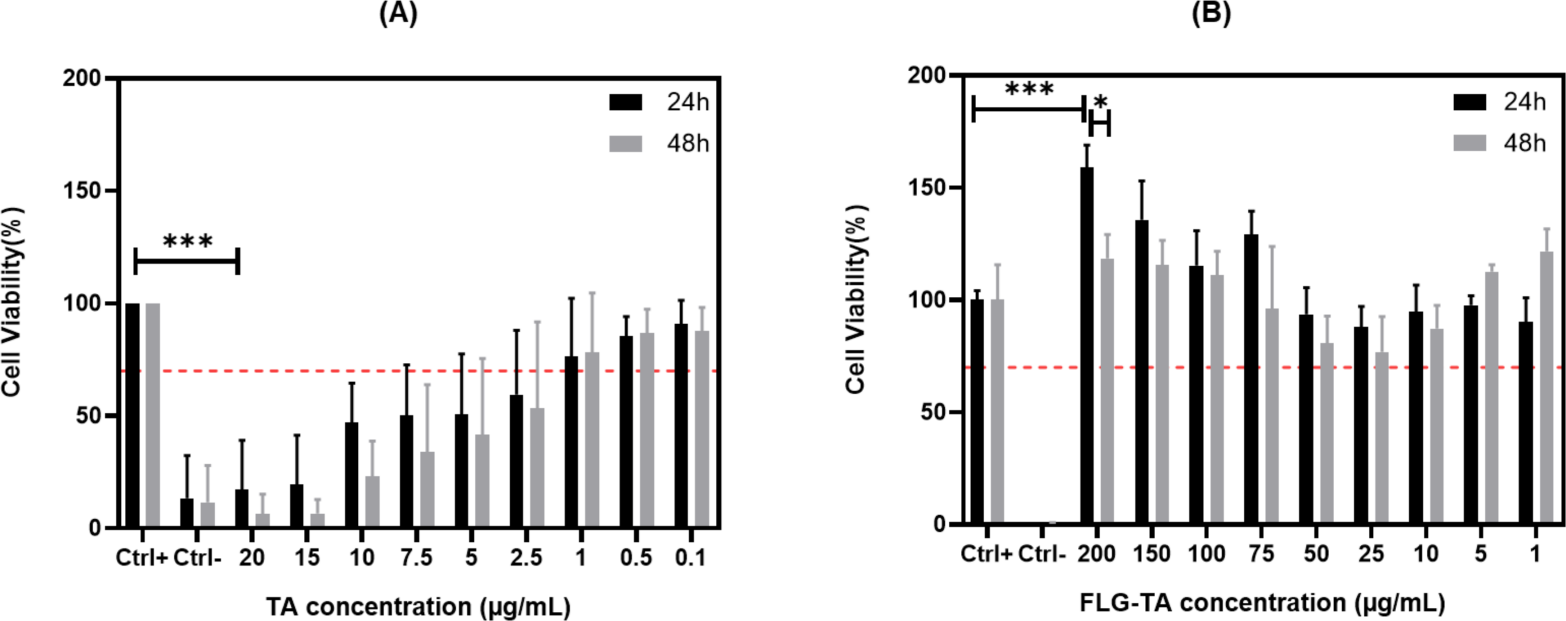
PDL cell viability was assessed using the Alamar Blue assay following exposure to either TA (A) or FLG–TA (B). Results are normalized to the positive control and shown as mean ± SD (*n* = 3). Statistical significance: *p ≤ 0.05, ***p ≤ 0.01 compared to control (Ctrl+), determined by Tukey's post-hoc test.

Previous findings demonstrated that TA adheres to the surface of graphene layers, with a portion of it retaining its antioxidant characteristics. Given TA’s significant antioxidant potential due to its phenolic functional groups [17], it was hypothesized that graphene layers rich in phenols could serve as a biocompatible interface for PDL cells. Thus, the cytotoxicity of the FLG–TA biocomposite was evaluated by measuring the metabolic activity of PDL cells. TA’s cytotoxicity on PDL cells was first tested. Note that in each experiment the amount of free TA acid per unit volume (Figure 3A) was compared with a concentration of the FLG–TA composite incorporating the same amount of TA (Figure 3B) remembering that almost all (85 %) TA added before the exfoliation was incorporated in or adsorbed on the FLG material. Figure 3A shows that the PDL cell viability drops below 70% within the concentration range of 2.5–20.0 µg·mL^−1^ for free TA molecules (***p < 0.001), demonstrating a significant cytotoxic effect of the polyphenol when not bound to graphene. This cytotoxicity is dose-dependent, increasing with the concentration of TA. This aligns with previous reports [17–18,24]. While TA exhibits antioxidant properties at low concentrations up to 20 µM [17,25], it can act as a prooxidant at high concentrations through metal ion chelation. Chromatin contains copper ions, which readily participate in redox reactions and bind strongly to DNA. These ions can form complexes with TA. Within cancer cells, for instance, this interaction can trigger ROS production and DNA damage [17]. However, when TA is complexed with graphene, a different scenario occurs as the FLG–TA composite shows no cytotoxicity up to a concentration of 200 µg·mL^−1^ (Figure 3B). These data altogether demonstrate that the addition of TA to FLG improves the cell viability compared to TA alone. The gradual increase in FLG–TA composite concentration is well tolerated by the cells, as they maintain their metabolic activity. This shift in cytotoxic behavior of TA when bound to graphene layers is noteworthy. The results also show that the observed slight increase in metabolic activity at concentrations of 200 μg·mL^−1^ is statistically significant (***p < 0.001) compared to the control group. Interestingly, this enhanced activity was followed by a notable decrease at 48 h (*p < 0.05). The initial increase in metabolic activity at 24 h could potentially be attributed to the TA coating on FLG particles enhancing cellular attachment. Previous research has demonstrated that cell adhesion may influence metabolic programming through regulation of adhesion molecules and glucose transport pathways [26–27]. Additionally, the molecular structure of tannic acid allows for potential enzymatic conversion into glucose derivatives via cellular metabolic pathways [28–29]. A previous study demonstrated that TA treatment enhanced glucose transport in 3T3-L1 cells through activation of insulin receptor phosphorylation and GLUT4 translocation [30]. These factors may have contributed to the observed enhancement in cellular metabolic activity at 24 h. The decrease in cell viability observed at 48 h may be related to changes in the stability of TA molecules within the culture medium, potentially resulting in dissociation of TA from FLG particles. The subsequent presence of unbound TA molecules may induce oxidative stress responses and compromise cellular viability.

Additionally, the material was tested for a longer period of 72 h. Results showed that cells maintained similar levels of metabolic activity at 72 h compared to measurements at 24 and 48 h. This confirmed that the used materials remained safe for cells over an extended period. These longer-term results can be found in Figure S6, Supporting Information File 1. To the best of our knowledge, no studies have reported this level of biocompatibility for FLG–TA within the dental context [4,31–32]. While earlier studies have examined the relationship between TA and carbon-based nanomaterials, they primarily focused on the adsorption mechanisms [15–16]. To date, no studies have explored how FLG–TA affects the biocompatibility of dental cells. The current research provides new insights into the biocompatibility of FLG–TA with PDL cells. We make the assumption that the cytotoxicity of TA present on the surface of FLG–TA with respect to the same amount of soluble TA is due to reduced mobility in the immobilized state. As shown in Figure S7, Supporting Information File 1, the LIVE/DEAD cell viability assay demonstrated that FLG–TA-treated cells maintained viability levels comparable to untreated controls. The assay revealed two distinct cell populations, namely, viable cells displaying intense green fluorescence (Calcein AM, ex/em: 495/515 nm) and non-viable cells exhibiting deep red fluorescence (SYTOX Deep Red, ex/mm: 660/682 nm). The proportion of viable cells remained consistently high across all tested FLG–TA concentrations. The high ratio of green-to-red fluorescence intensity observed in Figure S7, Supporting Information File 1, indicates robust cellular viability. These findings strongly align with previous metabolic activity results.

Because of strong molecular interactions during synthesis, FLG particles remain permanently bound to TA molecules, making it technically impossible to study isolated FLG. Therefore, our analyses focused on comparing TA and FLG–TA, which represent the only configurations possible under our experimental conditions.

### The antioxidant FLG–TA composite modulates ROS

Previous findings have shown that TA modifies the physicochemical and structural characteristics of GBMs [33]. The previously observed preservation of metabolic activity in FLG–TA likely stems from two main factors. First, the biocomposite’s ability to protect cells from ROS, which can originate from multiple sources including metal impurities (such as Fe, Ni, or Cu) present in the graphene structure, as well as from the intrinsic electronic properties of graphene itself. Indeed, graphene’s sp^2^ hybridization and delocalized π electrons can participate in electron transfer reactions, potentially leading to ROS generation [31,33]. While some TA molecules become less active upon adsorption on the graphene surface, those remaining accessible on the FLG–TA surface, particularly those with exposed chemical moieties oriented away from the graphene sheets, could maintain their free radical scavenging capabilities through multiple mechanisms, namely, electron transfer, where hydroxy groups donate electrons to neutralize free radicals, and hydrogen atom transfer, where hydrogen atoms from hydroxy groups are donated to stabilize ROS [17,34]. This retained antioxidant activity could play a crucial role in scavenging ROS, thus enhancing biocompatibility. Second, TA molecules potentially modify graphene’s surface structure, improving cellular adhesion.

**Figure 4:**
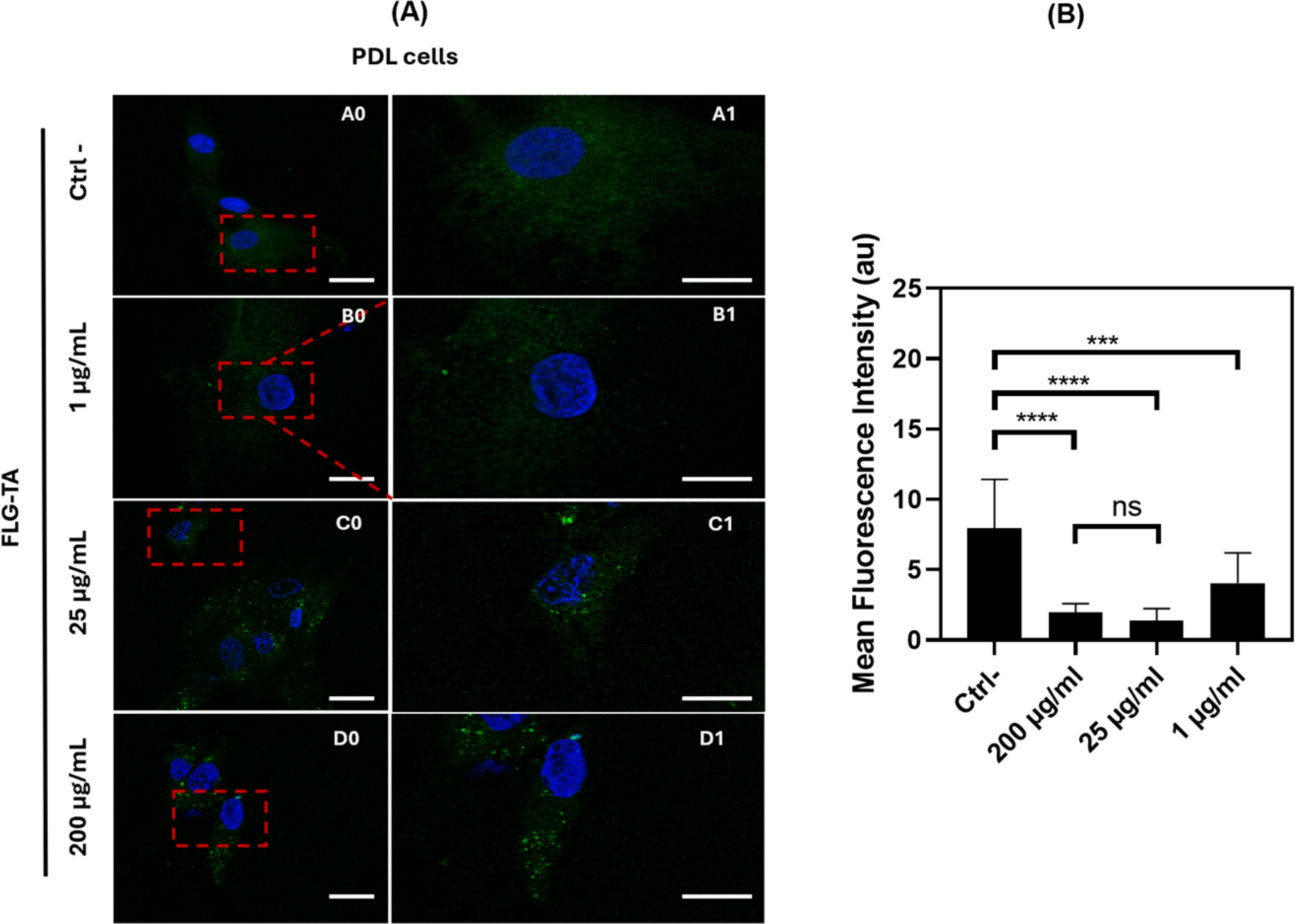
Reactive oxygen species (ROS) production in cells treated with FLG–TA. Confocal fluorescence images showing ROS levels after staining with CellROX (green) and Hoechst 33258 (blue). Scale bar: 20 µm (A). Quantification of ROS production following treatment with increasing concentrations of FLG–TA (B).

The effect of FLG–TA on ROS production in PDL cells was investigated. PDL cells were exposed to FLG–TA at concentrations of 1, 25, and 200 μg·mL^−1^, and ROS levels were measured using CellROX green stain. DMSO was employed as a control because of its established capacity to induce oxidative stress through enhanced ROS production and glutathione depletion, accelerating cellular necrosis [35]. This assay complemented the DPPH scavenging assay to assess the biocomposite’s cellular protective capabilities against ROS. Confocal microscopic studies indicated that cells displayed green fluorescence after FLG–TA exposure (Figure 4A). Fluorescence signal quantification showed intensities of 3.40 ± 0.35, 1.77 ± 0.97, and 1.71 ± 0.61 for FLG–TA treatments at 1, 25, and 200 μg·mL^−1^, respectively, compared to 5.13 ± 0.47 in DMSO-treated control cells (Figure 4B). These measurements correspond to ROS level reductions of 34%, 65%, and 67%, respectively, compared to DMSO controls. The decrease in the intracellular fluorescence is directly proportional to the amount of oxidative stress, thus elucidating the ROS inhibition by FLG–TA.

The UV–vis spectroscopic analysis (Figure S5, Supporting Information File 1) revealed a significant decrease in absorbance of the FLG–TA composite following exposure to DPPH solution, indicating oxidative modification of the material under ROS-mimetic conditions. The observed spectral changes suggest selective oxidation of phenolic moieties within the tannic acid component. Although this experimental model represents a simplified approximation of cellular ROS generation, it provides mechanistic insights into FLG–TA susceptibility to oxidative modifications under ROS-relevant conditions.

These results support our hypothesis that FLG–TA plays a crucial role in preventing ROS production and preserving biocompatibility. This research extends the findings of a prior study [36], which demonstrated the capacity of TA to alter the surface properties of carbon nanotubes and attributed the enhanced biocompatibility with respect to A549 cells to the antioxidant properties inherent to TA [36].

### FLG–TA promotes cellular adhesion

**Figure 5:**
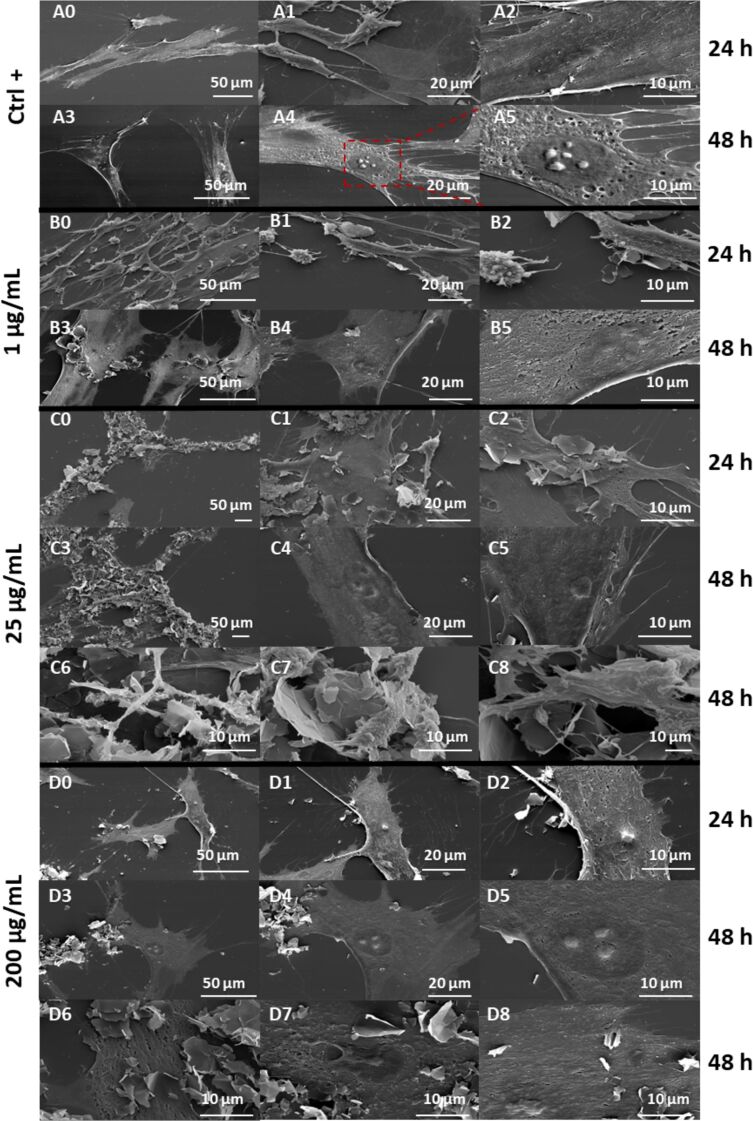
Scanning electron microscopy (SEM) images of PDL cells in contact with FLG–TA for 24 and 48 h, showing the progressive interaction between cells and the FLG–TA composite over time. Images are shown for control (A0–A5) and concentrations of 1 µg·mL^−1^ (B0–B5), 25 µg·mL^−1^ (C0–C8), and 200 µg·mL^−1^ (D0–D8).

**Figure 6:**
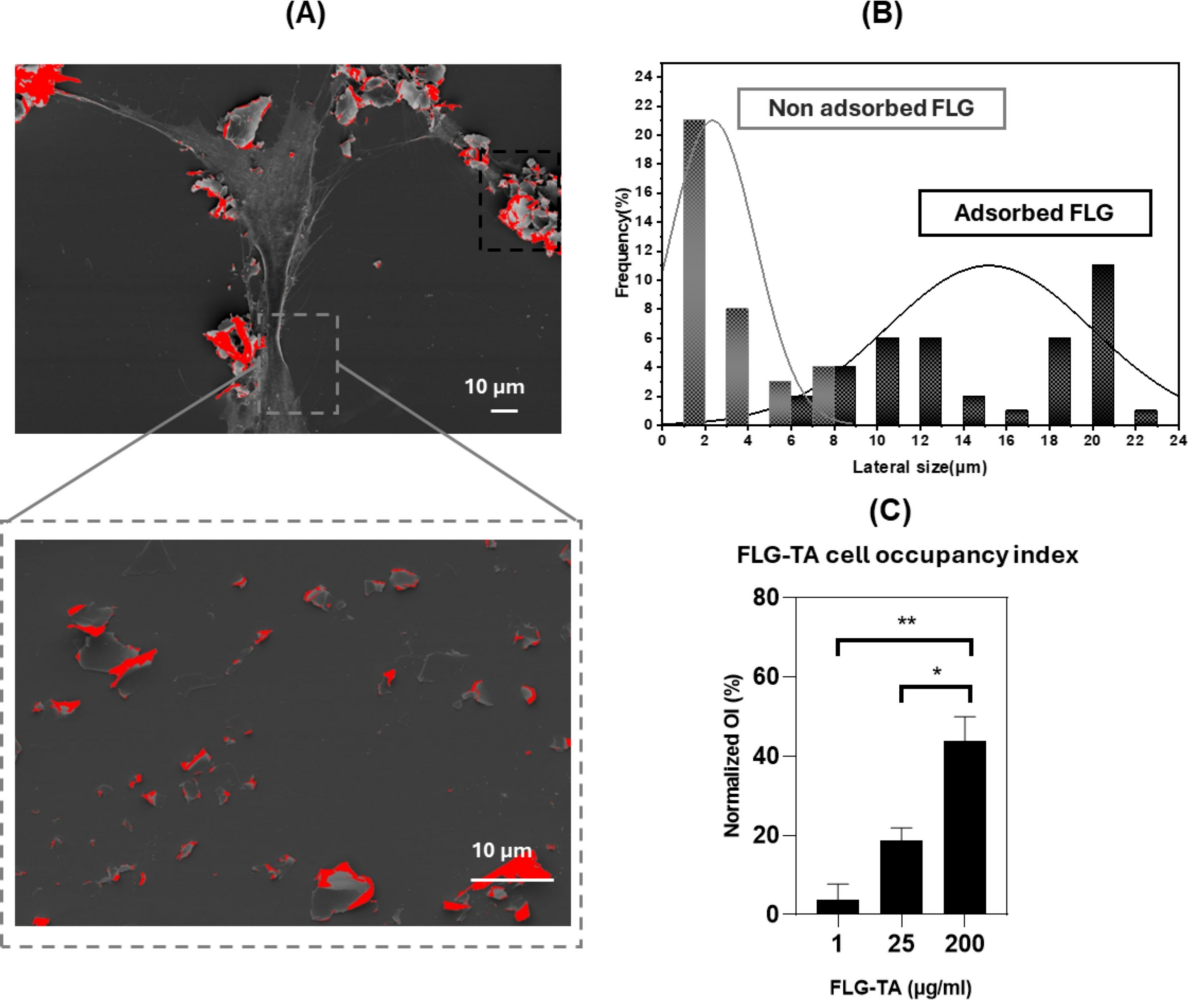
Scanning electron microscopy (SEM) micrographs representing FLG–TA and cell interaction (A), lateral size distribution of adsorbed graphene particles on cellular membranes (B), and graphene cell occupancy index (OI) (C). The occupancy index increases monotonically and significantly with rising FLG–TA concentration, reaching 43.7% at 200 µg·mL^−1^. The lateral particle size expands from 2 to 15 µm, indicating the formation of larger clusters on the cells’ surface.

Previous results indicated that TA molecules adsorb onto the graphene surface in the FLG–TA composite. These molecules maintain partial antioxidant properties, creating an interface that reduces ROS and provides protection against oxidative stress for PDL cells (Figure 4). To investigate whether TA adsorption affects cell adhesion properties beyond ROS reduction, PDL cell growth was monitored at 24 and 48 h following treatment with FLG–TA concentrations ranging from 1 to 200 µg·mL^−1^. Cellular morphology was examined using scanning electron microscopy (SEM, Figure 5), with untreated cells as controls. The occupancy index (OI) of FLG–TA in cell populations and the lateral dimensions of FLG–TA particles on cell membranes were measured (Figure 6). The obtained micrographs (Figure 5), revealed a complex 3D microenvironment that developed over 48 h, characterized by FLG–TA agglomerates and intricate cellular structures. The OI increased regularly and significantly with rising FLG–TA concentration, reaching a maximum of 43.7% at 200 µg·mL^−1^ (Figure 6C). In addition, the lateral particle dimensions expanded from 2 to 15 µm as the FLG–TA concentration increased (Figure 6B). Based on this observation, we can confirm that FLG–TA enhanced cellular adhesion while promoting cell proliferation. This finding aligns with earlier research indicating that TA coatings markedly enhanced the adhesion and proliferation of human liver cancer cells, specifically HepG2, on the PDMS substrate when compared to pristine PDMS [37]. TA may possibly play a role in cellular adhesion mechanisms, potentially through surface protein interactions. The cellular response on different graphene surfaces was previously studied, and it was demonstrated that substrate characteristics such as surface roughness, surface chemistry, and electronic properties can influence cell response [38]. The implications of these results are particularly significant in the context of dental biomaterials, where FLG–TA could potentially serve as a bioactive interface, enhancing cellular interactions and tissue integration.

### FLG–TA preserves chromatin integrity at concentrations up to 200 µg·mL^−1^

**Figure 7:**
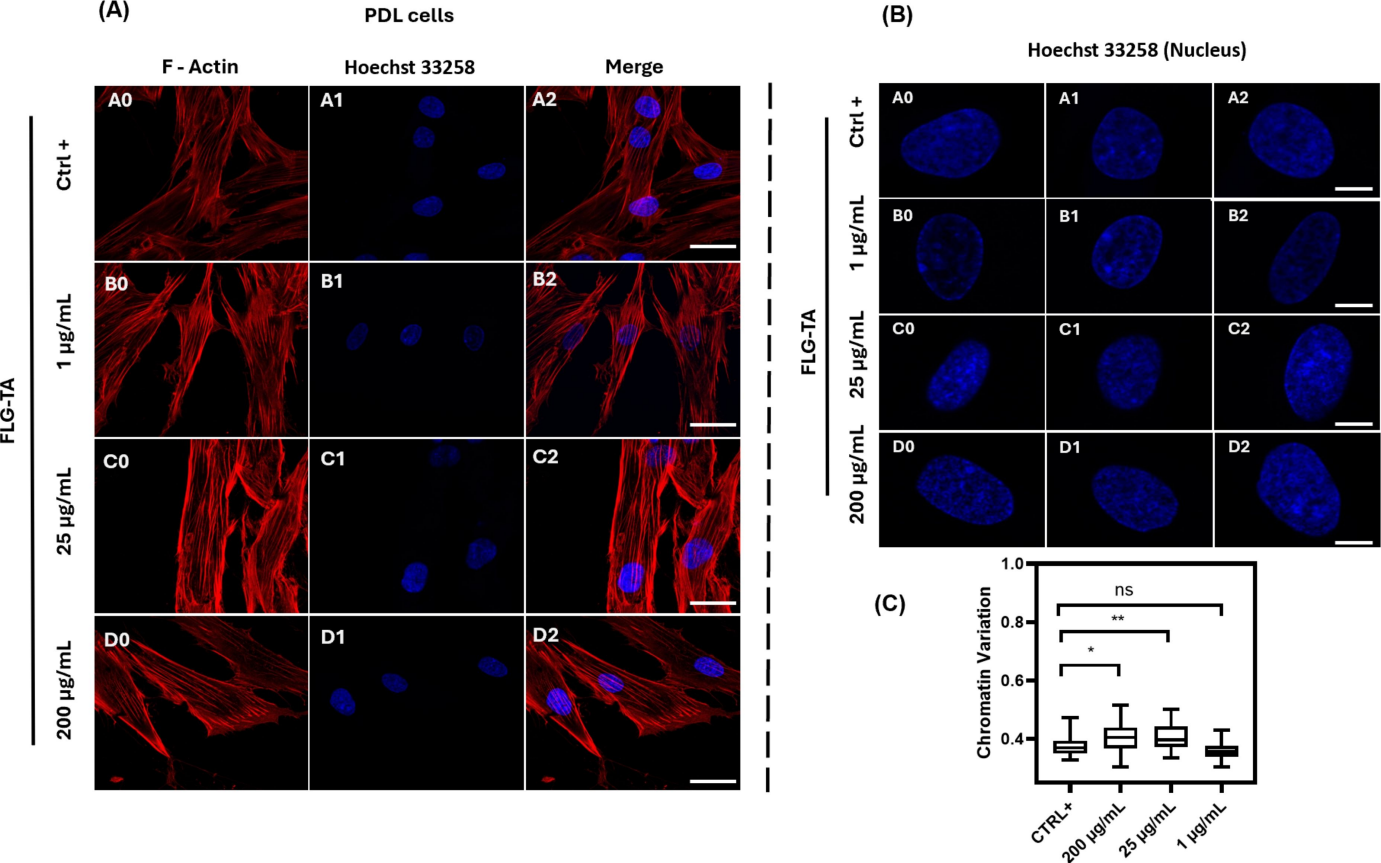
Representative confocal images of PDL cells following exposure to the FLG–TA material. The images display phalloidin-stained F-actin (red) and Hoechst 33258-stained DNA (blue), along with a merged view. Scale bar: 20 µm (A). Confocal images of PDL cells after contact with FLG–TA, highlighting Hoechst 33258-stained DNA (blue). Scale bar: 3 μm (B). Quantification of chromatin variation (CV) in the nucleus (percent). Values are presented as mean ± SD (100 < *n* < 170 nuclei from three independent experiments). Means labeled with * and ** are significantly different from the control (Ctrl+) at *p* ≤ 0.05 as determined by statistical tests followed by Dunn’s multiple comparison (C).

To validate the health status of PDL cells adhering to material surfaces as observed through SEM, we undertook additional confocal microscopy analyses concentrating on the actin cytoskeleton, which is a pivotal determinant of cellular structural integrity and adhesion capability, as well as overall cell viability [39–40]. The arrangement of actin filaments is recognized as a reliable metric for cellular health and substrate attachment quality [39–40]. Confocal micrographs (Figure 7A) demonstrated a well-preserved actin filament architecture across all evaluated FLG–TA concentrations (1, 25, and 200 µg·mL^−1^), thereby substantiating our prior assessments of metabolic activity. The maintenance of F-actin structural integrity, as visualized via phalloidin staining (red), offers compelling evidence supporting the biocompatibility of the material through sustained cytoskeletal organization. Figure S8, Supporting Information File 1, provides additional representative micrographs at low magnification.

After examining cellular adhesion, we investigated whether FLG–TA affects the chromatin structure. DNA comes together with histone proteins to create chromatin, which is essential for processes such as replication, transcription, and repair [41–43]. The level of chromatin compaction can affect all these processes. Changes in chromatin structure play a key role in maintaining proper cellular functions such as cell growth, cell cycle advancement, and cell death [43]. Disorganized alterations in chromatin structure can result in abnormal gene activity [43]. Different harmful substances can use various mechanisms to alter DNA structure and impact the integrity of the cell nucleus [43]. To study how chromatin is organized in PDL cells after exposure to different amounts of the FLG–TA material, we measured the chromatin variation (CV) parameter. The coefficient of variation represents a quantitative methodology for evaluating nuclear DNA signal heterogeneity. This analytical approach has demonstrated utility in measuring alterations in DNA compaction following drug treatments or genetic modifications that influence chromatin architecture [43]. PDL cells exposed to 200 and 25 µg·mL^−1^ FLG–TA show CV values of 0.36 ± 0.06 and 0.32 ± 0.05, respectively, compared to the values of PDL cells exposed to 1 µg·mL^−1^ FLG–TA (0.30 ± 0.03) and CTRL+ (0.3 ± 0.4) (Figure 7C). The very small increases in chromatin compaction for conditions of 200 and 25 µg·mL^−1^ FLG–TA, compared to those of 1 µg·mL^−1^ PLG-TA and CTRL+, should have no impact on cell fate because for all conditions the CV values are around 0.4. It was shown that in mouse fibroblasts grown on a graphene monolayer on a glass substrate, chromatin decompaction was induced [44]. Our results differ, probably due to our different FLG synthesis procedure with TA and its mode of PDL cells exposure. Under the tested conditions, our results indicate that FLG–TA, at concentrations up to 200 µg·mL^−1^, has no genotoxic effect on PDL cells.

## Conclusion

A bioactive few-layered graphene–tannic acid biocomposite was successfully synthesized via a simple method. By integrating antioxidant TA molecules into the FLG surface, the biocomposite dynamically mitigated ROS, demonstrating no cytotoxicity to PDL cells up to 200 µg·mL^−1^ while promoting cellular adhesion and maintaining chromatin integrity. Overall, with its satisfactory biocompatibility, FLG–TA potentially offers new insights as a novel biomaterial for dental applications.

The addition of TA to the FLG surface enhances both physicochemical and biological properties. This advantageous combination expands the potential uses of the biocomposite across biomedical applications and dentistry. In future work, we will incorporate the FLG–TA composite into various dental biomaterials, such as sealer resins and calcium silicate cements, to improve their mechanical and physicochemical properties.

## Experimental

### Reagents and materials

Tannic acid (*M*_W_ = 1701.20 g·mol^−1^) and Hoechst 33258 were obtained from MERCK Sigma-Aldrich Reagent (Darmstadt, Germany). α-MEM cell culture medium was purchased from Gibco (Thermo Fisher Scientific, Waltham, MA, USA). Fetal bovine serum (FBS) and phosphate-buffered saline (PBS) were acquired from Dominique Dutscher (Brumath, France). The resazurin cell viability assay kit (Alamar Blue) was sourced from Biotium (Fremont, CA, USA). Other reagents and solvents were procured from Thermo Fisher Scientific (Waltham, MA, USA). Deionized water used throughout this work was obtained using a Milli-Q water system (Merck Millipore, Burlington, MA, USA).

### FLG–TA synthesis

Graphite (1 g) and tannic acid (0.1 g) were added to 1 L of distilled water in a vial. The mixture was sonicated using an ultrasonic probe (Branson Digital Sonifier 550, 110 W, 40 Hz) for 2 h in an ice-water bath under continuous stirring. The resulting suspension was then allowed to settle for 24 h.

### Physicochemical characterizations

SEM images were obtained using a high-resolution scanning electron microscope (JEOL JSM 5600, France) operating at 10 kV with a working distance of 10 mm. FLG sheets were individually dispersed in water, and a few drops of the resulting solution were pipetted onto clean silicon wafer substrates. The substrates were then air-dried and mounted on aluminum pins using double-sided carbon tape. Corresponding SEM images were subsequently captured.

The S/TEM characterization was performed using a JEOL2100F microscope outfitted with a probe corrector for spherical aberrations. All bright-field and dark-field STEM images were acquired at an accelerating voltage of 200 kV.

Raman analysis was performed using a LabRAM ARAMIS confocal microscope spectrometer equipped with a CCD detector. The spectra were recorded in the range of 200–3200 cm^−1^ using a 532 nm/100 mW emission from a YAG laser source with an MPC600 PSU quantum laser to excite the samples.

The XPS measurements were conducted in an ultrahigh vacuum setup (base pressure 5 × 10^−9^ mbar) comprising several interconnected chambers. The analysis chamber features a RESOLVE 120 MCD5 hemispherical electron analyzer and a dual-anode source (Mg/Al). Samples were mounted on holders using conductive double-sided carbon adhesive tapes. XPS measurements utilized the monochromatic Al Kα line at 1486.6 eV. An initial survey scan identified elements present in each sample, followed by high-resolution scans of major element lines. Survey and high-resolution spectra were recorded using constant pass energy mode with energies of 100 and 20 eV, respectively. Data analysis was performed using Casa XPS 2.3.23 software.

To quantify free and adsorbed TA, the previously prepared FLG–TA solution was filtered through a 0.22 μm PVDF membrane until a clear solution was obtained. The absorbance of the filtrate was immediately measured using a UV–vis spectrophotometer at 275 nm (*A*_275_). Free tannic acid concentrations *C* (in µg·mL^−1^) were determined using the equation *A*_275_ = 0.4131*C* − 0.0004 (*r*^2^ = 0.9972) derived from the calibration curve. The total quantity of tannic acid adsorbed on the surface of the FLG was then calculated from the difference between the initial and the final TA concentrations and reported per unit mass of carbon-based material. Attempts to evaluate the FLG–TA particle size and zeta potential in their diluted suspensions were done using a Nano ZS device from Malvern, operating in the backscattering mode.

The free radical scavenging assay was carried out to assess the antioxidant properties of both free and adsorbed TA on the surface. A 10^−4^ M DPPH solution was prepared in absolute ethanol, and 1 mL of this solution was mixed with 1 mL of TA solution at various concentrations (0.006–4.000 μg·mL^−1^). The absorbance was then measured at 517 nm against a blank sample (ethanol). Lower absorbance values in the reaction mixture indicate higher DPPH free radical scavenging activity. A standard curve was established by combining different concentrations of TA with DPPH, allowing for the determination of DPPH concentration scavenging capacity expressed as concentration in the reaction medium through a linear regression analysis (*r*^2^ = 0.9672). The capability to scavenge the DPPH radical was calculated using the formula


[1] $$\text{scavenging activity (\%)}=\frac{A-B}{A}\cdot 100\%,$$

where *A* represents the absorbance of DPPH and *B* represents the absorbance of the DPPH and TA combination. A similar experiment was conducted to assess the antioxidant activity of FLG–TA deposited on quartz in the form of a film. In this parallel examination, the deposited film was put in contact with a 10^−4^ M DPPH solution in the absence of light, followed by the absorbance measurement at 517 nm.

### Biological assays

#### Isolation and culture of PDL cells

PDL cells were isolated from human alveolar ligament obtained from extracted human teeth. This study was conducted with approval from French Ministry of Higher Education and Research (CODECOH DC-2021-4812). PDL cells were isolated from premolars extracted from healthy donors (*n* = 4, age range 20–30 years) attending private surgery for orthodontic reasons. Information about the research project was provided to patients during pre-operative appointments, and oral informed consent and non-opposition to the use of their biological waste for research reasons was obtained. Before use, the cells’ phenotypic characteristics were validated through flow cytometry, evaluating the expression of CD90, CD73, CD34, CD45, HLA-DR, CD105, and CD11b [45]. The cells were cultured in a specialized medium (Gibco™ α-MEM, Thermo Fisher Scientific, Waltham, MA, USA) supplemented with 10% FBS and 1% penicillin–streptomycin (Dominique Dutscher, Bernolsheim, France). Cells from passages 3 to 7 were used for experiments.

#### Metabolic activity

The Alamar Blue (AB) assay was used to quantify the metabolic activity of PDL cells in direct contact with FLG and TA solutions by detecting the oxidation–reduction rate of AB [46]. This assay has gained significant popularity as a common method for examining cytotoxic effects induced by various test compounds [46]. Briefly, cells were seeded at a density of 1 × 10^5^ cells/well (passages 3 to 7) in a 96-well plate with α-MEM essential medium supplemented with 10% FBS, 100 µg·mL^−1^ penicillin, and 100 µg·mL^−1^ streptomycin (Dutscher). The cells were treated with FLG and TA in fresh medium at concentrations ranging from 0 to 200 µg·mL^−1^, along with a negative control containing 100 µL of dimethyl sulfoxide (DMSO). Cells were incubated at 37 °C with 5% CO_2_ for 24 h reaching 80% confluence. After 24 and 48 h of exposure, the medium was removed, cells were washed twice with PBS and incubated with a 10% AB solution in complete α-MEM medium for 2 h under 5% CO_2_ at 37 °C in the dark. Supernatants were then transferred to a black 96-well plate and fluorescence was measured using an excitation wavelength of 530 nm and an emission wavelength of 590 nm on a Varioskan spectrophotometer (ThermoFischer, USA). Cytotoxicity was determined as the percentage of cell viability relative to untreated control cells. Non-seeded wells with AB mixed with different concentrations of graphene served as background controls to ensure that FLG alone did not interfere with the assay. Each experimental condition was run in triplicate, and experiments were replicated at least three times.

#### LIVE/DEAD assay

A complementary LIVE/DEAD assay was adopted using a Thermo Fisher Kit with Calcein AM and SYTOX™ Deep Red nucleic acid stain. Cells treated with different concentrations of FLG–TA were incubated with a working solution containing 2 μM Calcein AM and 0.25 μM SYTOX Deep Red for 30 min. After the cells were washed with PBS to optimize signal-to-noise ratio, they were imaged using epifluorescence microscopy with FITC filter set for live cells (green fluorescence) and deep red filter set for dead cells (red fluorescence).

#### Oxidative stress

Reactive oxygen species (ROS) levels were evaluated using CellROXgreen (Molecular Probes Inc., Eugene, OR). PDL cells at a density of 1 × 10^5^ cells/mL were cultured on glass coverslips (CML, France). PDL cells were then treated with various concentrations of FLG–TA or left untreated for 24 h at 37 °C. After treatment, cells were stained with 5 µM CellROXgreen probe and Hoechst 33258 for 30 min at 37 °C, then washed with PBS [47]. The cells were subsequently fixed and mounted using the VectaShield medium. Confocal laser scanning microscopy (LSM 710, Zeiss, Germany) with a 40× objective was used for imaging, using excitation/emission wavelengths of 485/530 nm. DMSO-treated cells served as a control group. All procedures were carried out in darkness to prevent light exposure. The ImageJ software was used to analyze fluorescence intensity in microscopy images. Results are expressed as the percentage reduction in ROS levels compared to the control group.

#### Cell morphology

To assess potential changes in cell morphology induced by FLG–TA, scanning electron microscopy (SEM) was performed on treated and untreated cells after 24 and 48 h of direct contact. Cells were cultured on 12 mm diameter glass coverslips (CML, France) in a 24-well plate and exposed to FLG–TA concentrations ranging from 1 to 200 µg·mL^−1^. Adherent cells were washed twice with PBS and fixed in 2.5% glutaraldehyde (Euromedex, France) in 0.05 M sodium cacodylate buffer (Euromedex, France) at pH 7.4 for 2 h. Cells were then washed three times with cold 0.175 M sodium cacodylate buffer for 10 min each. The samples then underwent dehydration through a cold graded ethanol series (30%, 50%, 70%, and 95%) for 7 min each, followed by two 5-minute rinses in cold absolute ethanol and one 5-minute rinse in room-temperature absolute ethanol. Finally, samples were dehydrated with hexamethylenedisilazane for 15 min and air-dried in a fume hood for 24 h and coated with a gold–palladium alloy using a Hummer JR sputtering device (Technics, CA, USA), creating a 12.5 nm thick gold layer (50 s at 20 mA sputter current). SEM observations were conducted using a SEM (FEI Company, Eindhoven, Netherlands, 10 kV). All analyses were performed in triplicate. To calculate the cell occupancy index, SEM images were acquired at 2000× magnification, with five random fields captured per sample, analyzing approximately five cells per field. These images were then processed through ImageJ software using thresholding to distinguish between cells covered with FLG–TA particles and vacant areas of cells. The cell occupancy index (OI) was calculated by measuring the ratio between the area covered by FLG–TA at the surface of adherent cells and the total available surface area of cells. This was expressed as a percentage using the formula in Equation 2. All measurements were performed in triplicate and the results were expressed as mean ± standard deviation.


[2] $$\text{OI (\%)}=\frac{\text{area covered by FLG}-\text{TA}}{\text{total surface area of cells}}\cdot 100\%$$

#### Evaluation of chromatin architecture

To measure the chromatin variation parameter (CV), cells were cultured on 12 mm diameter glass coverslips (CML, France) in a 24-well plate. They were fixed and permeabilized for 15 min in a solution of 4% paraformaldehyde (Electron Microscopy Sciences) and 0.1% Triton X-100 diluted in 1× PBS. F-Actin was then labeled using the Actin Red 555 probe (Thermo Scientific), and DNA was stained with Hoechst 33258 (Sigma Aldrich). Samples were mounted in VectaShield medium. Stained nuclei were observed using a confocal microscope (LSM 710, Zeiss, Germany) with a 63× PL APO (1.4 NA) objective. DNA fluorescence was detected after excitation at λ = 405 nm, using a bandpass emission filter from 406 to 486 nm. The total nuclear volume was determined by optimizing the slicing distance [41]. CV was calculated using a MATLAB script based on Irianto et al.’s method [42], which uses a Sobel gradient-based edge detection technique to assess nuclear edge density and identify condensed chromatin regions. Cell nuclei were segmented using ImageJ's “crop and split” approach before CV calculation.

### Statistical analysis

Statistical analysis was conducted using GraphPad Prism software version 8.0 (GraphPad Software, San Diego, CA, USA). For continuous responses, the data were represented as mean ± standard deviation derived from at least three independent experiments with triplicate observations. To assess the normality of the collected numerical data, the D’Agostino and Pearson and Shapiro–Wilk normality tests were utilized. Statistical significance was evaluated using one-way analysis of variance (ANOVA), followed by Dunnett’s multiple comparison test. A *p*-value smaller than 0.05 was considered statistically significant.

## Supporting Information

File 1 Additional figures and tables.

## Data Availability

Data generated and analyzed during this study is available from the corresponding author upon reasonable request.
