# Supplementary material for: Colloidal few layered graphene–tannic acid preserves the biocompatibility of periodontal ligament cells
Source: Beilstein J Nanotechnol. 2025 May 20;16:664–77. doi: 10.3762/bjnano.16.51 (PMC12117215; doi:10.3762/bjnano.16.51)
Supplement: File 1 — Additional figures and tables. [file Beilstein_J_Nanotechnol-16-664-s001.pdf]

BEILSTEIN JOURNAL OF NANOTECHNOLOGY

Supporting Information

for

Colloidal few layered graphene–tannic acid preserves the biocompatibility of periodontal ligament cells

Teissir Ben Ammar, Naji Kharouf, Dominique Vautier, Housseinou Ba, Nivedita Sudheer, Philippe Lavalle and Vincent Ball

Beilstein J. Nanotechnol. **2025,** *16,* 664–677. doi:10.3762/bjnano.16.51

Additional figures and tables

License and Terms: This is a supporting information file under the terms of the Creative Commons Attribution License (https://creativecommons.org/licenses/by/4.0). Please note that the reuse, redistribution and reproduction in particular requires that the author(s) and source are credited and that individual graphics may be subject to special legal provisions.
The license is subject to the *Beilstein Journal of Nanotechnology* terms and conditions: (https://www.beilstein-journals.org/bjnano/terms)

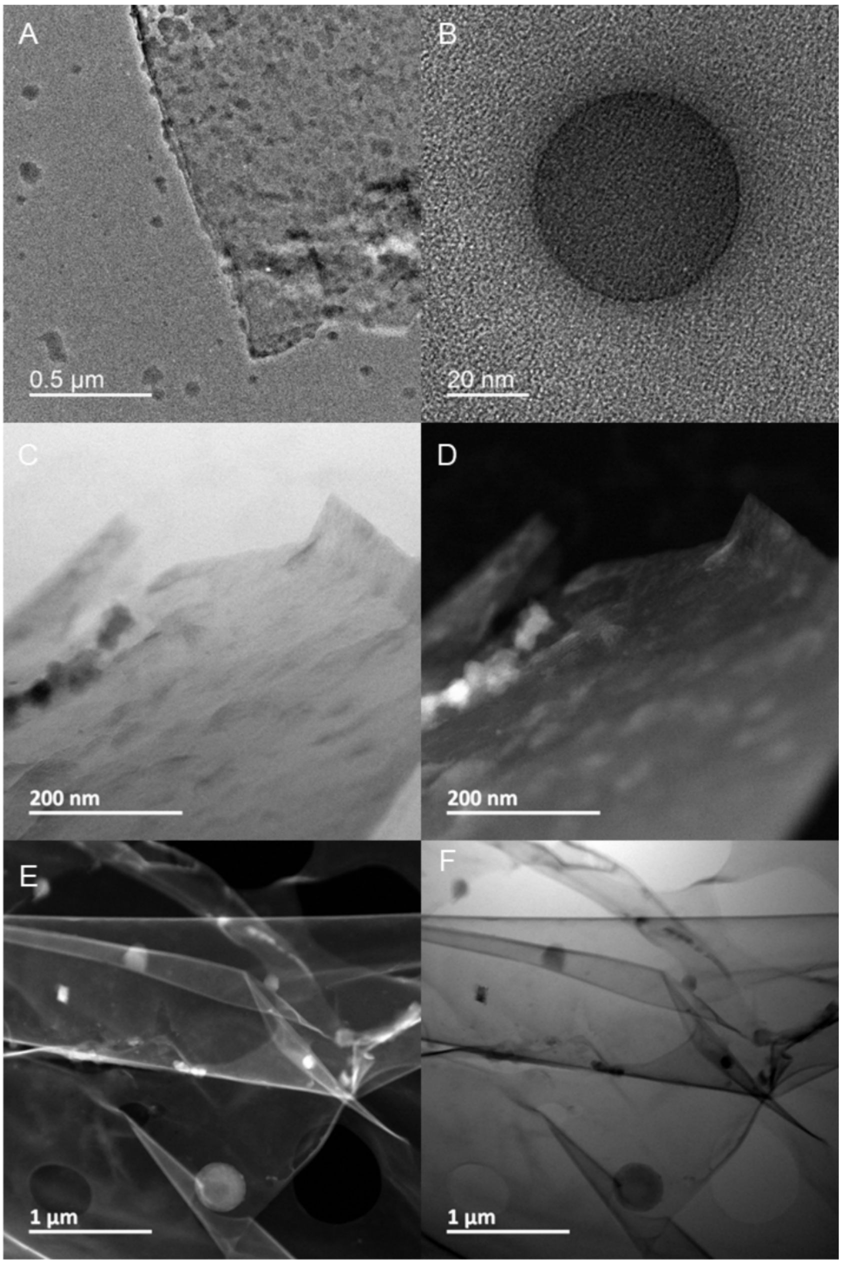

Figure S1: Representative transmission electron microscopy (TEM) micrographs of FLG layers obtained after liquid-phase exfoliation of graphite assisted by TA (A). Structure of spherical granules present on top of layers (B). BF-STEM (C, F) and DF-STEM (D, E) images of FLG sheets and particle distribution on the surface. BF: bright field; DF:dark field.

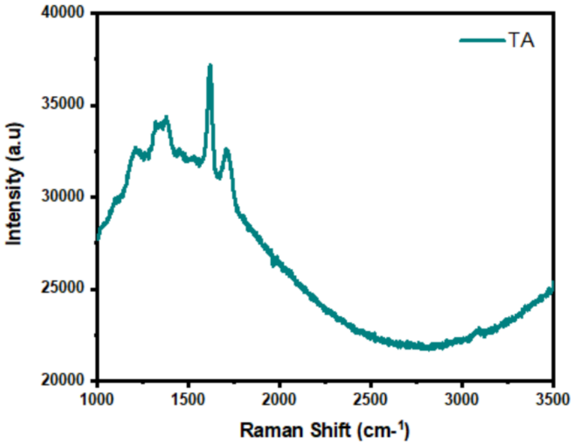

Figure S2: Raman spectrum of tannic acid.

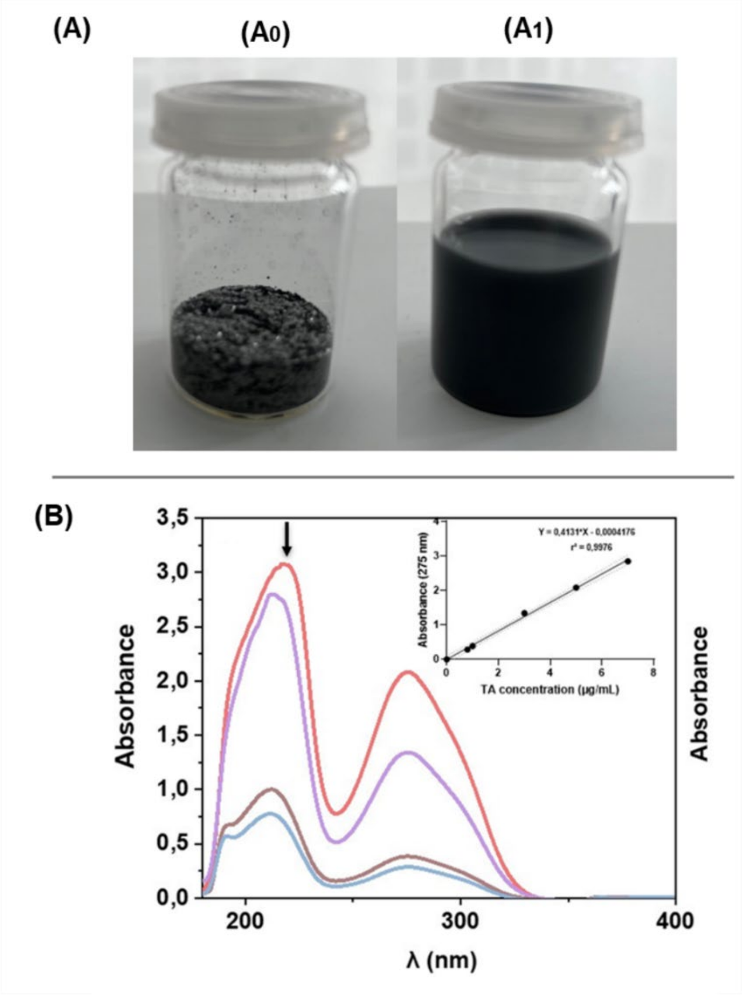

Figure S3: Picture of the initial graphite (A0) and FLG–TA colloid after ultrasonication and sedimentation (A1). UV–visible spectra of TA with different concentrations (0–8 µg·mL^{-1}) in the wavelength range of 180–400 nm. The inset represents the linear calibration curve between the concentrations of TA and the absorbance at 274 nm (B).

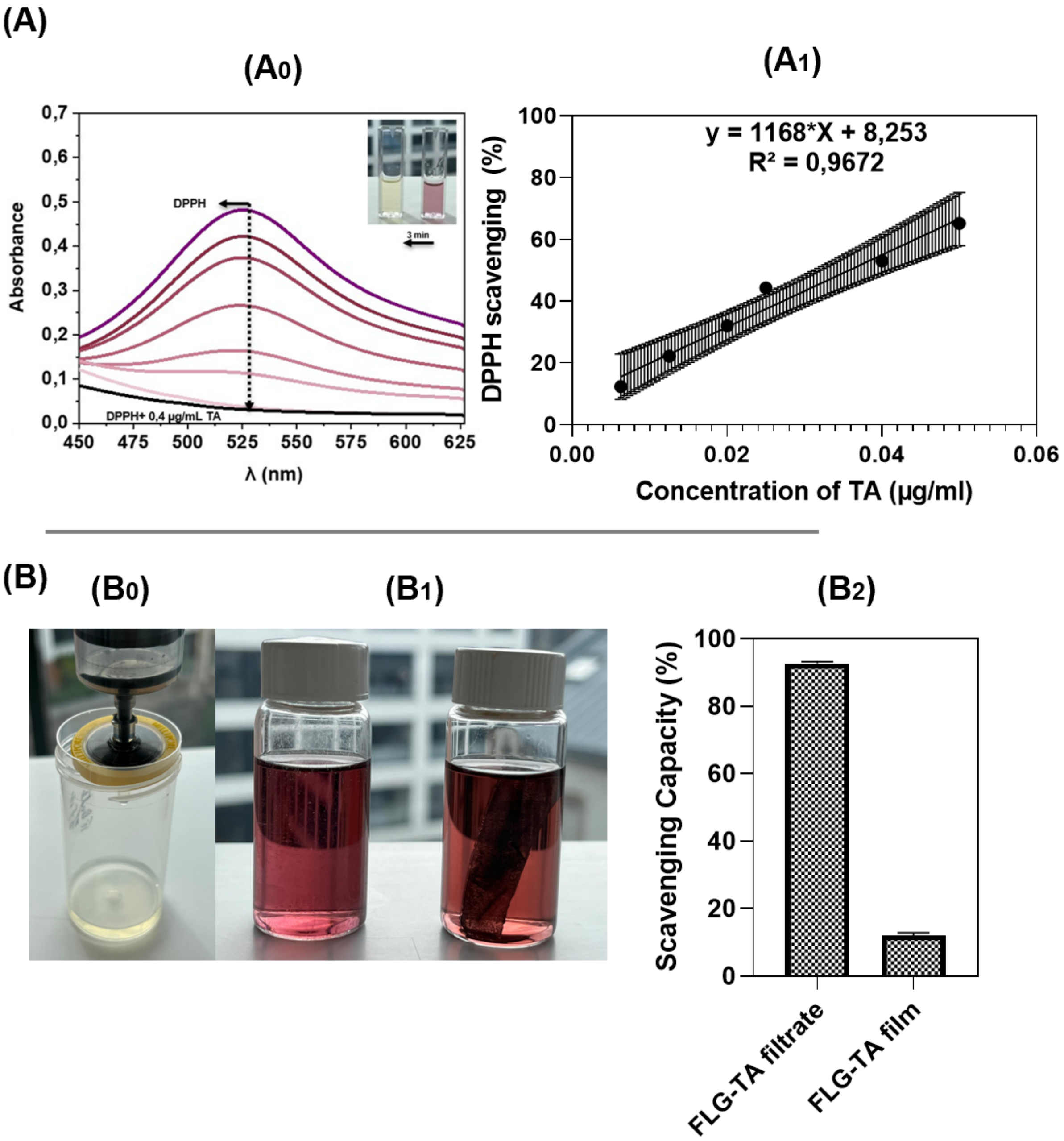

Figure S4: Determination of TA's ability to scavenge stable DPPH free radicals by monitoring the intensity of the DPPH radical absorption peak at 517 nm (A0). Calibration curve between TA concentrations (µg·mL^{-1}) and respective DPPH scavenging capacity (%) at 517 nm (A1). Pictures showing the DPPH scavenging capacity of FLG–TA filtrate (B0) and FLG–TA film (B1), and their respective experimental DPPH scavenging capacities (%) (B2).

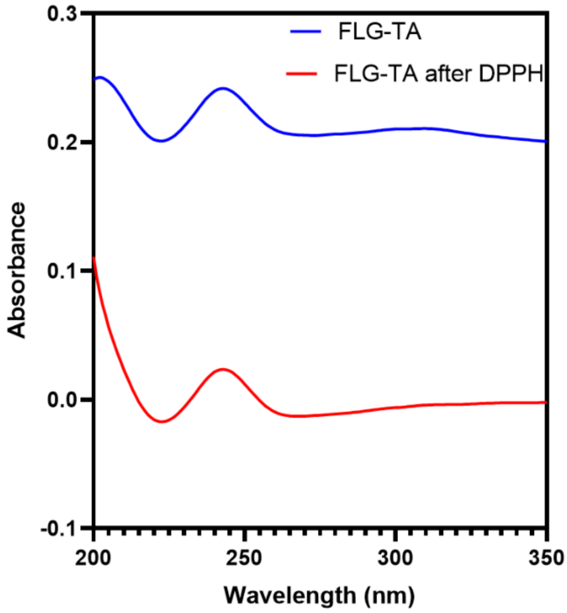

Figure S5: UV–visible absorption spectra of FLG–TA films before and after exposure to DPPH radical solution (10^{-4} M, 30 min incubation), demonstrating changes in material stability under oxidative conditions.

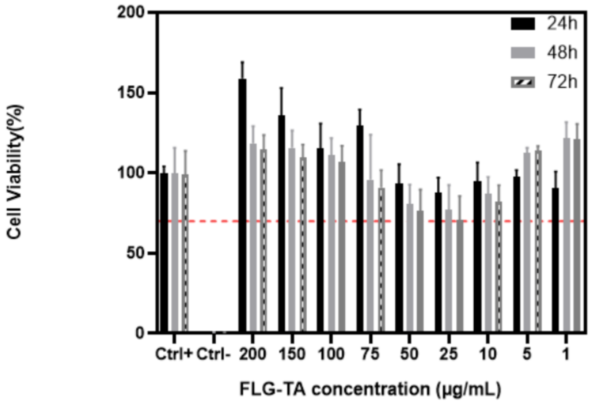

Figure S6: Cell viability assessment of PDL cells exposed to FLG–TA. Alamar Blue assay was performed at 24, 48, and 72 h timepoints. Data are presented as mean ± SD (n = 3), normalized to positive control.

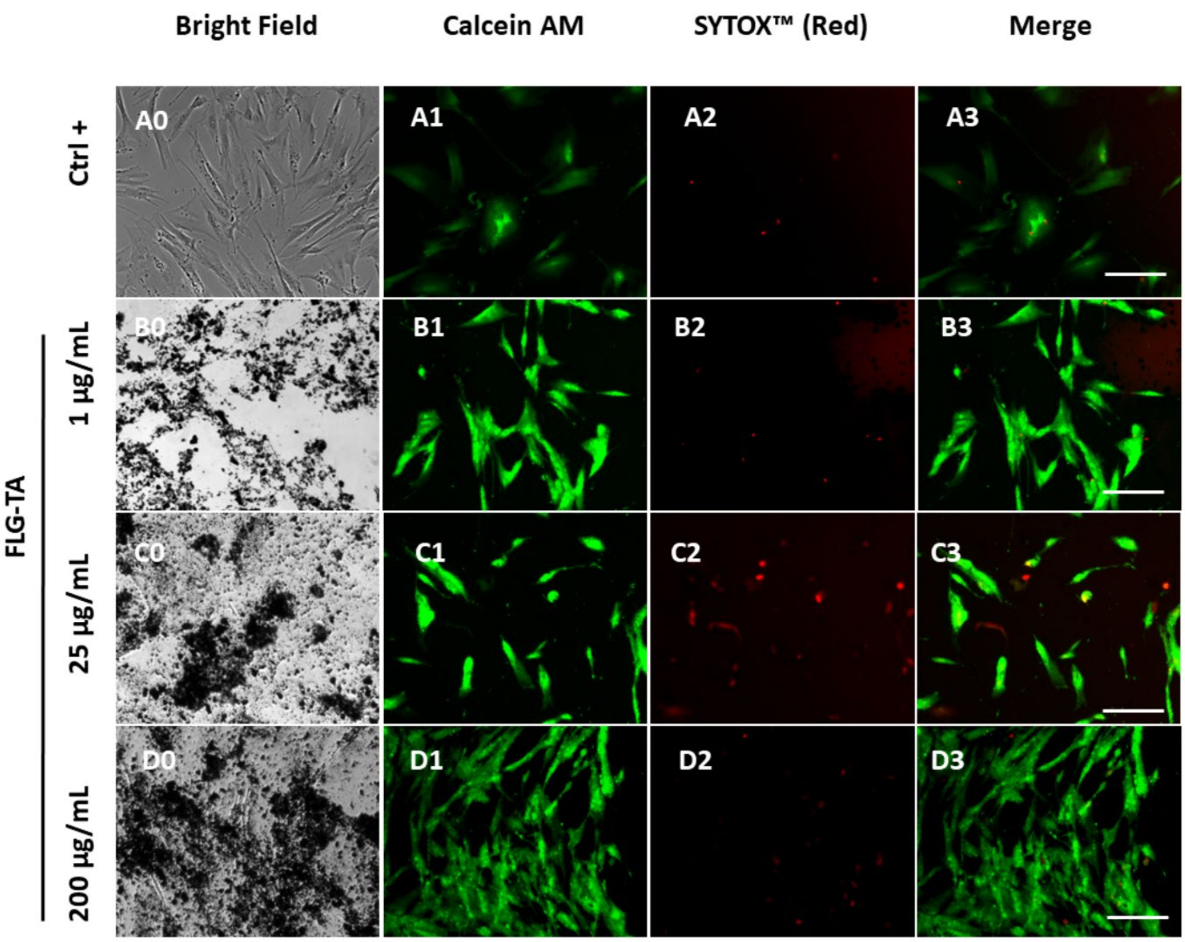

Figure S7: Representative fluorescence microscopy images showing cell viability assessment using LIVE/DEAD assay. Live cells exhibit green fluorescence (Calcein AM, ex/em: 495/515 nm) while dead cells show deep red fluorescence (SYTOX™ Deep Red, ex/em: 660/682 nm). Scale bar: 60 µm.

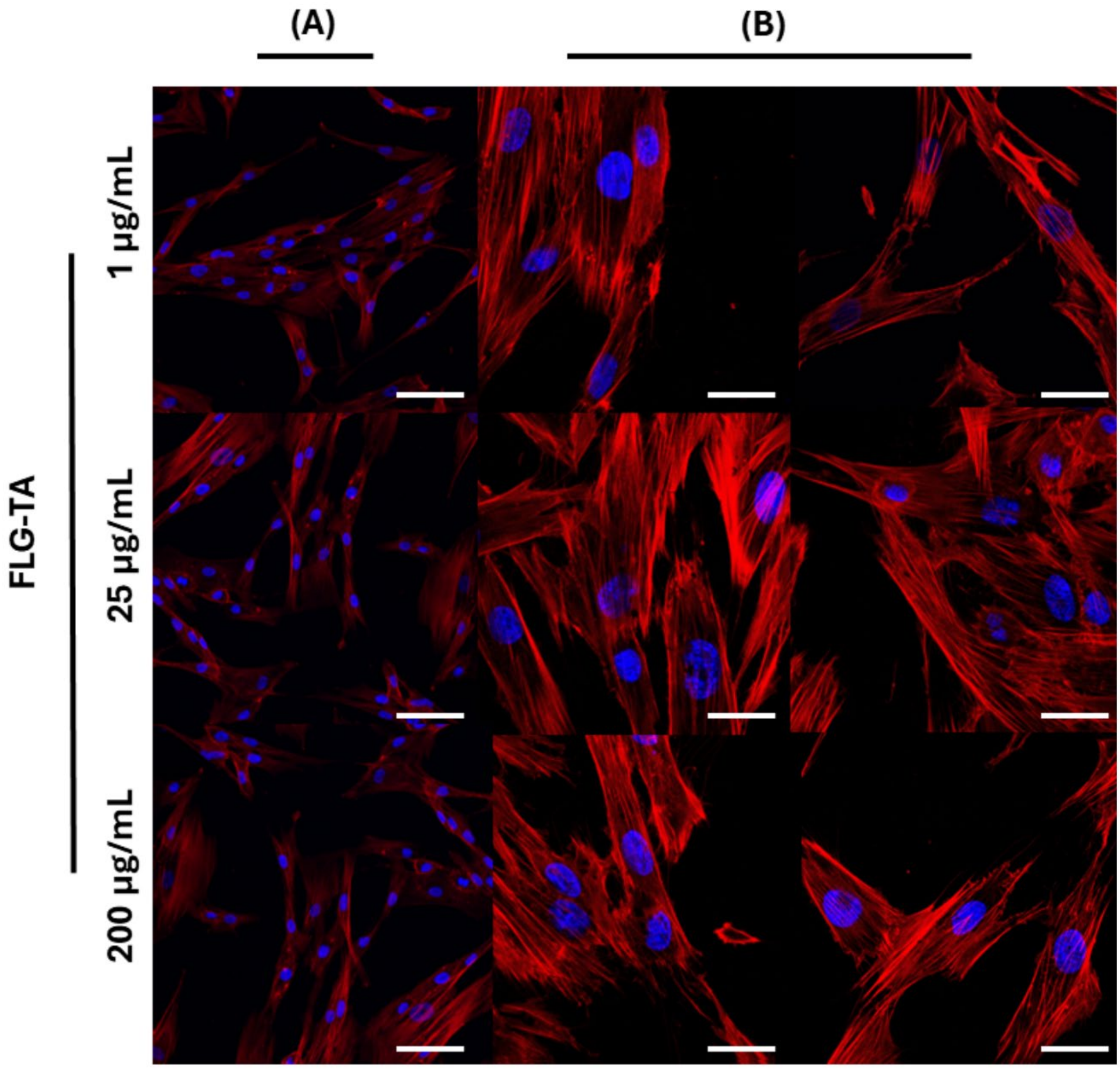

Figure S8: Additional representative merged confocal images of PDL cells exposed to FLG–TA at 1, 25, and 200 μg·mL^{-1}, showing phalloidin-stained F-actin (red) and Hoechst 33258-stained DNA (blue). (A) Low magnification, scale bar: 60 μm and (B) higher magnification, scale bar: 20 μm.

Table S1: XPS data of graphite and FLG–TA including the contribution of different carbon moieties obtained by deconvolution of C 1s, and O-to-C ratio obtained from the area of O 1s and C 1s peaks.

Sample	XPS analysis (atom %)					O 1s/C 1s
	C–C, C=C	C–OR/defects	C=O	COOR	π–π*	
Graphite	80.98	7.31	4.74	3.32	3.66	0.17
FLG–TA	73.06	13.06	5.94	3.75	4.06	0.45
